# Increased time-to-pregnancy is associated with domestic work in South Africa

**DOI:** 10.1186/s12978-016-0224-y

**Published:** 2016-09-06

**Authors:** Braimoh Bello, Dick Heederik, Danuta Kielkowski, Kerry Wilson

**Affiliations:** 1 Division of Environmental Epidemiology, Institute for Risk Assessment Sciences, Utrecht University, Utrecht, Netherlands; 2 Epidemiology and Surveillance Unit, National Institute for Occupational Health, Johannesburg, South Africa

**Keywords:** Fertility, Fecundity, Time-to-pregnancy, Occupation, Domestic workers, South Africa

## Abstract

**Background:**

The effects of female occupational exposures on fecundity have not been evaluated in South Africa. The aim of this study was to assess the effects of three specific occupational groups on time-to-pregnancy (TTP).

**Methods:**

This cross-sectional study collected data, by means of a questionnaire, on 1210 women representative of a South African population, and sought information on: TTP for the most recent pregnancy, time-specific information on maternal factors and occupational exposures, as well as some paternal factors. Occupational exposure groups were determined using employment profile prior to the pregnancy. In the risk analysis, domestic workers and teachers were compared to administrative staff. Accidental and unplanned pregnancies were excluded from the analysis and participants who were never pregnant were censored. Discrete-time Cox regression models were built to estimate fecundability ratios (FR).

**Results:**

The median TTP in administrative workers, domestic workers and teachers was 4, 12 and 3 months respectively. After adjusting for a number of potential confounders, TTP was significantly related to occupation at the time of pregnancy attempt. Compared to administrative workers, domestic workers had a significantly lower per-cycle probability of conception (adjusted FR = 0.53; 95 CI 0.32–0.88). The per-cycle probability of conception in teachers compared to administrative workers was not significantly different (adjusted FR = 1.14; 95 CI: 0.75–1.72).

**Conclusion:**

Domestic work was associated with prolonged TTP. Working as a domestic worker in South Africa may affect fecundity.

## Plain English summary

Time to pregnancy (TTP) is the number of months it takes a woman to conceive. This duration varies from couple to couple and reflects variation in *fecundity*, the biological capacity to conceive. TTP has been used in many developed countries to determine how occupation affects fecundity. But in Africa, no study has investigated this. This study sought to determine the association between occupation and time-to-pregnancy and it is the first in the continent to do so.

This cross-sectional study conducted in a South African population collected data from 1210 women on TTP for their most recent pregnancy, the work they were doing before the pregnancy and other factors. Most women in the community were administrative workers, domestic workers and teachers. So the TTP of these three groups were compared.

Compared to administrative workers, domestic workers had lower fecundity. The average (median) TTP in administrative workers, domestic workers and teachers was 4, 12 and 3 months respectively.

The study showed that working as a domestic worker in South Africa may affect fecundity. These results may be true for many African settings and highlight the need for more research into occupation and fertility in Africa. There is a need to consider policies that will protect women from reproductive harm in the workplace, especially those in vulnerable jobs.

## Background

TTP is the number of non-contraceptive menstrual cycles (months) it takes a couple to conceive. It is a continuum that covers the entire distribution of waiting times, from zero months to several years [1–3]. TTP is used to measure fecundity, the biological ability to conceive. An increased TTP reflects decreased fecundity and may be due to injury to the reproductive system. Although fecundity and fertility are sometimes used interchangeably because fecundity plays a role in fertility, they actually refer to different constructs. Fecundity is the biological (physiological) capacity to reproduce. Fertility, on the other hand, is a demographic concept which refers to the actual production of live offspring, the number of live births a woman has had. While fecundity is a purely biological phenomenon, fertility is determined by biological (fecundity) and behavioural factors such as contraceptive use and pregnancy planning [2, 4]. Infertility is clinically defined as TTP longer than 1 year [5–7].

Domestic workers and administration clerks comprise a sizeable proportion of the South African female workforce. With about one million women employed in each group, they are the two largest specific occupational groups in the country, accounting for 15 and 17 % respectively of all employed women [8, 9]. Domestic workers are exposed to a number of agents that have been documented to affect time-to-pregnancy. For example, chlorine, surfactants, formaldehyde, perchloroethylene (PERC) and ammonia commonly present in household cleaning products, soaps, air fresheners, and scouring powders, have been shown to be associated with increased time-to-pregnancy and pregnancy loss [10, 11].

Despite the significant number of domestic workers employed in South Africa and the potential health hazards they face, there have been very few studies evaluating health outcomes among domestic workers in the country. The few studies on domestic workers have majorly focused on working conditions, power-relationship and workers’ rights [12–15]. Global research into domestic work and health outcomes is equally scanty. A search of the literature found no studies that have evaluated fertility in domestic workers in any setting.

In a previous TTP study carried out in South Africa, increased TTP was associated with employment status. After adjusting for potential confounders and excluding the role of biases, including the infertile-worker-effect, women who were employed at the time of pregnancy attempt had a longer TTP than those who were unemployed [16]. Interestingly, majority (40 %) of the employed women in the study were domestic workers. Due to the small number of participants in the study, further analysis by specific employment type could not be conducted and conclusions were cautious. That preliminary study generated the hypothesis that domestic work in South Africa is associated with reduced fecundity. Furthermore, no studies have evaluated the association between specific occupational groups and time-to-pregnancy (TTP) in South Africa.

This study aimed to evaluate TTP among domestic workers, teachers and administrative workers. It is the first study to compare TTP among occupational groups in South Africa and Africa in general.

## Methods

### Study design and setting

This study is based on a cross-sectional reproductive health survey conducted in a South African population in 2008. The study had a number of objectives including to describe the distribution of reproductive health outcomes – planned pregnancy, TTP, spontaneous abortion and stillbirth – in the population and to investigate associations between specific reproductive health outcomes and occupational groups. This analysis evaluated the association between TTP and occupation, using the three most dominant occupational groups identified in the survey.

Detailed methodology of the study, including the reliability of the questionnaire tool and the distribution of reproductive health outcomes for the study population, has previously been reported [17]. In brief, the study was a population-based cross-sectional survey of reproductive age (18–49 years) women in Potchefstroom, a South African town. Unlike most South African towns which have predominantly one or two races, Potchefstroom was chosen because its racial distribution was similar to that of the general South African population. Systematic sampling, stratified by race and ward, was carried out to obtain a representative sample of reproductive age women in the population. Data on TTP, occupation at pregnancy attempt and other co-variates were collected by means of a validated questionnaire previously piloted in the same population [16].

### Assessment of TTP

A questionnaire measuring TTP and work history provides a useful tool for assessing the effect of specific occupation on fecundity [1]. Repeatability analysis of the tool used in this study showed high reliability for TTP and other variables measured in the population [17].

TTP information for the most recent pregnancy was sought in order to achieve good recall of information and high data quality. TTP was measured as the number of months it took the participant to fall pregnant. TTP can only be available for women who planned to have a pregnancy. Therefore, prior to asking the TTP question, participants were asked: 1) whether they planned (expected) the pregnancy; 2) whether they used contraceptives before the pregnancy; and 3) for those who used contraceptives, whether they fell pregnant while using contraceptives. Participants were also asked whether they sought medical intervention to fall pregnant and after how many months of trying, if they did.

### Assessment of occupation and covariates

A key consideration for retrospective ascertainment in TTP studies is to ensure that the exposures were present at pregnancy attempt i.e. before pregnancy. Cross-sectional studies that collect TTP data by retrospective ascertainment approximate retrospective cohort studies as the focus is on exposures before pregnancy occurred [1]. In this study, questions on occupation and other factors were asked with reference to the period before pregnancy i.e. at pregnancy attempt. A section of the questionnaire sought data on occupational characteristics including the industry they worked in, the company name, as well as their job title. Data were also collected on a number of covariates including age, income, smoking status, alcohol use, gravidity, parity, frequency of sexual intercourse and presence of a chronic disease.

### Statistical analysis

Statistical analyses were performed using STATA version 13.0 software (Stata Corp, Texas, USA). Using data on type of industry, workplace and job title, three occupational groups were generated and compared: administrative (office) workers, domestic workers and teachers. The administrative group comprised of office workers who reported their job title at the time of pregnancy as clerks, administrative staff or assistants, librarian, cashiers, secretary and receptionists. Domestic workers reported their job as domestic workers or cleaners while teachers reported theirs as teachers or lecturers. The reference group was administrative workers.

For TTP analysis, all women with TTP values were included: accidental and unplanned pregnancies were excluded. TTP values were only available for participants who planned their pregnancies and reported the number of months it took them to fall pregnant. Those who were never pregnant were censored at the time of survey.

Appropriate descriptive statistics were reported and Kaplan-Meier survival curves were used for distribution of TTP by occupation. The log-rank test was used to determine if TTP distribution differed significantly across occupational groups. For median TTP and TTP categories, Kruskal-wallis test and Chi-squared test were used. Discrete-time Cox regression models, which handled the TTP data (number of months taken to fall pregnant) as a discrete scale, were used to estimate hazard ratios of fecundability (fecundability ratios (FR)) and 95 % confidence intervals. The FR represents the per-cycle probability of conception in one occupational group relative to a reference occupational group. Since fertility is a desired outcome, unlike in morbidity or mortality studies, a FR less than 1 is undesirable, implying reduced fecundity. The multivariable cox model was built to estimate the association between occupation and fecundity adjusting for potential confounders. The selection of confounders relied on knowledge of the literature and empirical evidence. Causal links between the potential confounders, occupation and TTP was the primary criterion used in the selection of confounders. Using knowledge of the field, a pool of potential confounders which met the criteria for confounding was selected for adjustment. Furthermore, statistical analysis was used to identify other confounders in this population. For this, potential confounders which caused a 10 % change in estimate were adjusted for.

## Results

### Characteristics of participants at pregnancy attempt

**Fig. 1:**
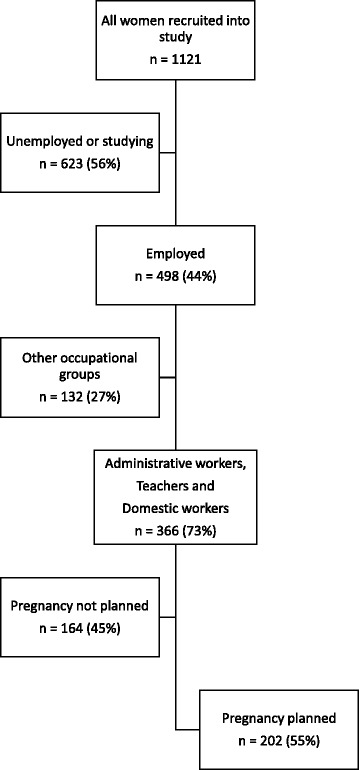
Flow chart showing the total number of women recruited into the study. The chart shows the number of women recruited into the survey and those included in the analysis. The final sample included the 202 women in the three major occupational groups in the population who had a TTP value for their most recent pregnancy

As shown in Fig. 1, 1121 women were recruited into the survey, reflecting a response rate of over 90 %. Of the 1121 women, 498 (44 %) were employed at the time of their last pregnancy, reflecting the low levels of employment in the community, which is generally the same for the entire South Africa. Of those employed, 366 (73 %) belonged to three major occupational groups which were analysed in this study. Of these 366 women, 202 (55 %) women planned their most recent pregnancy and had a TTP value.

This analysis included the 202 women in the three major occupational groups – administrative workers, domestic workers and teachers – who reported TTP for their most recent pregnancy. The characteristics of the women at the time of pregnancy attempt are shown in Table 1. The table shows that the mean age of administrative workers and domestic workers was similar (28 years) while that of teachers was slightly higher (30 years), although the difference was not statistically significant. Race is an important variable in South Africa, often acting as a proxy for socio-economic and contextual disparities in the population. The majority of administrative workers were White women (61 %) while 20 % were Black women. Also, majority of teachers were White women (81 %). In contrast, about three quarters of domestic workers (74 %) were Black women. There were no significant differences in prior contraceptive use, coital frequency and mean gravidity across the groups.

**Table 1:** Distribution of TTP covariates at the time of pregnancy attempt, by occupational group

Variable	Administrative workers	Domestic workers	Teachers	*P*-value
	*N* = 106	*N* = 31	*n* = 65	
Mean age in years (sd)	28 (4)	28 (6)	30 (4)	0.1369
Race (%)				<0.01
Black	20	74	9	
White	61	0	86	
Coloured	14	26	2	
Indian	5	0	3	
Education (%)				< 0.01
Primary or less	6	54	0	
Some secondary	8	42	0	
Completed secondary	42	4	5	
Tertiary	43	0	95	
Monthly income in ZAR^a^ (%)				< 0.01
Less than 2500	10	97	3	
2500–4999	21	3	8	
5000–9999	39	0	30	
10000 and above	30	0	59	
Smoked (%)	15	26	5	0.016
Had a chronic disease (%)	9	29	9	0.009
Previously used contraceptive (%)	81	67	83	0.189
Coital frequency (%)				0.343
Almost daily	43	47	46	
Once a week	22	25	18	
Less than once a week	21	21	18	
Don’t know	14	6	18	
Mean gravidity	1.2	1.4	1.2	0.6133
Mean partner’s age	31	30	32	0.5021
Partner smoked (%)	37	67	28	<0.01

^a^1 South African Rand (ZAR) is approximately 0.1 USD

### Time-to-pregnancy by occupational group

Table 2 shows the planned pregnancy rate and TTP by occupational group.

**Table 2:** Planned pregnancy and TTP category by occupational group

Variable	Administrative workers	Domestic workers	Teachers	*P*-value
	*N* = 106	*N* = 31	*n* = 65	
Planned pregnancy (%)	58	32	76	< 0.01
Median TTP (IQR)	4 (2–11)	12 (5–36)	3 (2–9)	< 0.01
TTP Category (%)				< 0.01
1–3 months	45 %	19 %	60 %	
4–6 month	22 %	19 %	12 %	
7–12 months	14 %	23 %	8 %	
More than 12 months	19 %	39 %	20 %	

**Fig. 2:**
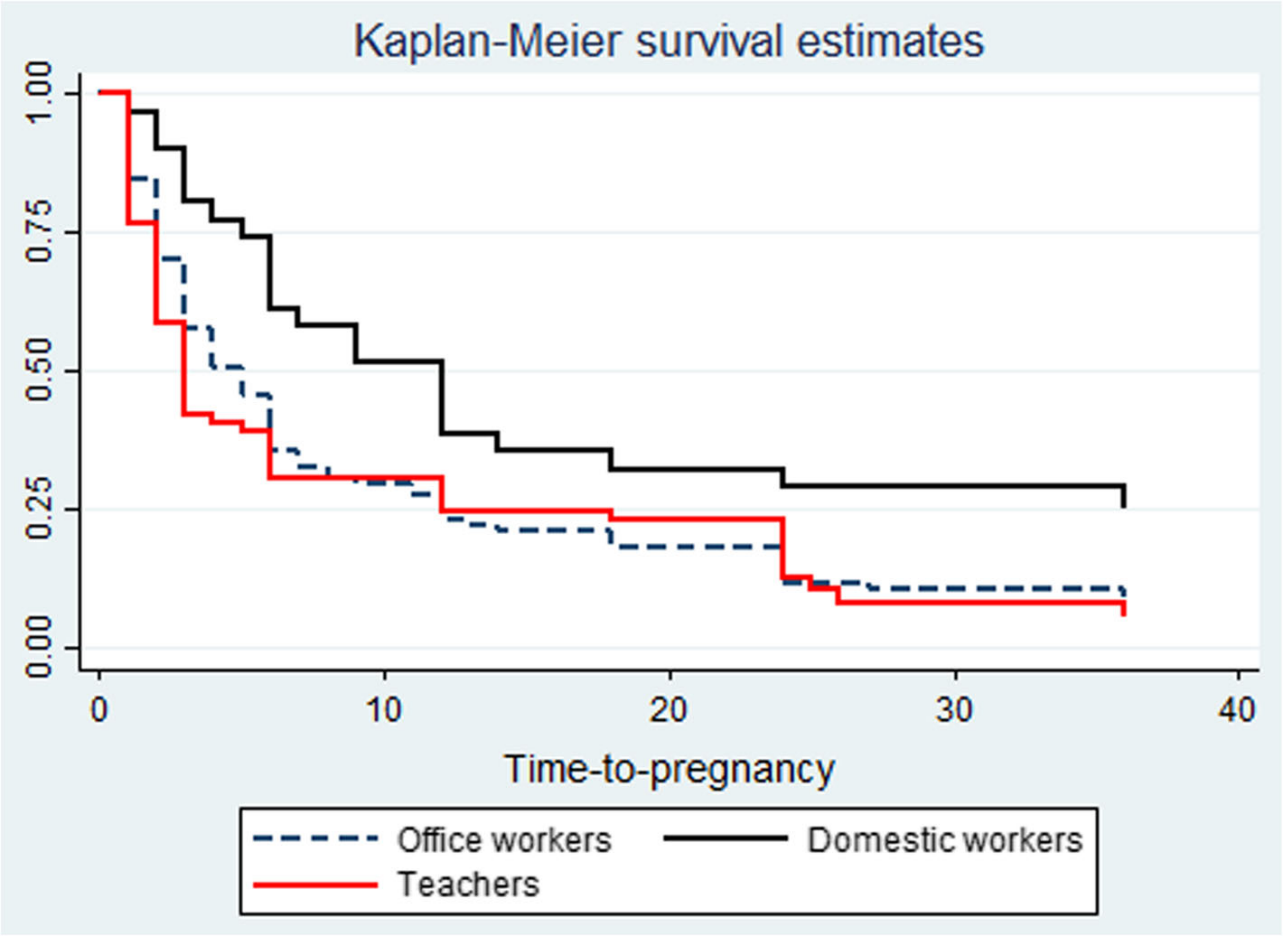
Kaplan Meier survival curve showing time-to-pregnancy occupational group. Kaplan Meier survival curve showed that domestic workers had longer time-to-pregnancy than administrative workers and teachers. After 12 months, about 40 % of domestic workers were still not pregnant, as compared to 20 % of administrative workers and teachers

Planned pregnancy differed significantly by occupational group: it was lowest (32 %) for domestic workers and highest (76 %) for teachers (*p* < 0.01). Median TTP was longest for domestic workers (12 months), followed by administrative workers (4 months) and the teachers (3 months). Accordingly, the proportion of women who took longer than 12 months to fall pregnant was highest for domestic workers (39 %). The log-rank test showed that there were significant differences in TTP distribution by occupational group (*p* < 0.01) – Fig. 2.

The univariable proportionate hazard model showed that domestic workers, compared to administrative workers, were significantly less likely to fall pregnant (unadjusted FR = 0.52; 95 CI 0.33–0.81). This result remained significant after adjusting for six important potential confounders (adjusted FR = 0.59; 95 CI 0.35–0.99), confirming that the association between domestic work and TTP was not confounded by these variables (Table 3). There was no significant difference in TTP between teachers and administrative staff (adjusted FR = 1.10; 95 CI 0.78–1.56).

**Table 3:** Unadjusted and adjusted fecundability ratios (FR) of the association between occupation and TTP

Occupational group	Unadjusted fecundability ratio (95 % CI)	Adjusted fecundability ratio^b^ (95 % CI)
Administrative workers	1	1
Teachers	1.08 (0.74–1.57)	1.14 (0.75–1.72)
Domestic workers	0.46 (0.29–0.72)^a^	0.53 (0.32–0.88)^a^

^a^Significant at 0.001

^b^Adjusted for maternal age, race, gravidity, smoking, presence of a chronic disease and coital frequency

## Discussion

This is the first study to investigate the effect of any occupation on TTP in Africa and the first to do so for domestic workers in any setting. The results showed that domestic workers took significantly longer time to fall pregnant when compared to administrative workers, suggesting that domestic work may have negative effect on fecundity.

The finding is supported by a previous TTP study in the same population, which suggested that that fecundity may be significantly reduced for domestic workers. The study found increased TTP for employed women when compared to unemployed women [16]. Although the sample size for that study was too small to allow for occupation-specific analysis, a description of the occupation of the employed women showed that majority (40 %) were domestic workers.

It is well known that TTP data collected by means of a questionnaire provide valid estimates for how long it takes a woman to conceive [1, 18]. Occupation and other co-variates at the time of pregnancy attempt are unlikely to have been misclassified as women are well able to remember their reproductive events and the context around them [18]. Also, this study was based on the most recent pregnancy – 56 and 76 % of most pregnancies were in the last 5 and 10 years respectively. TTP recall of up to 20 years have been validated in Europe [1, 18, 19], and the high validity and reliability of the questionnaire used in this study has been previously reported [17]. However, the possibility of certain biases in TTP studies cannot be entirely excluded [1, 6, 20]. Therefore, we discuss the alternative explanations and plausible causal explanations for the finding. First alternative explanations, due to the potential role of confounding and bias, are discussed. Then plausible causal explanations for the results are discussed.

### Confounding

The crude analysis showed a decreased fecundity for domestic workers in comparison to administrative workers (unadjusted FR = 0.46; 95 % CI 0.29–0.72). The results remained valid even after adjusting for six maternal confounders (adjusted FR = 0.53; 95 % CI 0.32–0.88). Furthermore, additional adjustment for partner age and smoking status did not change the effect (adjusted FR = 0.53; 95 % CI 0.31–0.92).

The number and types of confounders adjusted for in the study can be considered sufficient to get refined estimates of independent association between fecundity and occupational group, but the possibility of residual confounding cannot be entirely ruled out. An important potential confounder that was adjusted for in this study was coital frequency. This is because in South Africa, many domestic workers stay at the home of their employer during the week, only returning to their own homes on weekends, thereby reducing their coital frequency. The hypothesized effect of occupation in TTP studies is on fecundity. Therefore, any effect due to reduced coital frequency should be controlled for. However, the adjusted analysis showed that coital frequency was not a confounder in this study. This is because in this population, almost all domestic workers lived in the same district as their employers and went to work daily from home.

### Bias

#### Planning bias

Further analysis was conducted to determine if planning bias played a role in this finding because the proportion of women who planned their pregnancy and reported a TTP value differed significantly by occupation. Domestic workers were less likely to plan their pregnancy than administrative workers and teachers. The effect of planning bias was assessed by assigning a TTP value of 0 to women who did not plan their pregnancy. The analysis did not show that planning bias had an effect on the results as the effect remained the same – adjusted FR = 0.53; 95 % CI 0.32–0.88. This is supported by the observation elsewhere that non-planners do not necessarily have higher fecundity than planners [21].

#### Time-trend bias

This population-based TTP study, as opposed to pregnancy based TTP studies, captured the entire TTP distribution from 1 month (high fecundity) to many years (low fecundity) for those who were still trying to fall pregnant at the time of the survey. Including women who were still trying to fall pregnant at the time of the survey has the benefit of eliminating fertility bias which can occur in pregnancy-based TTP studies. However, participants’ characteristics can change over time, for example they might change jobs and behaviours, as the waiting time increases. To evaluate the role of time-trend bias, we censored TTP at 14 months. The effect of domestic work remained similar (adjusted FR: 0.62, 95 % CI: 0.36–1.06) confirming that time-trend bias did not play a role in this finding.

#### Medical intervention (infertility treatment) bias

Medical intervention bias is unlikely to have played a role in this study. Respondents were asked if they sought medical intervention to become pregnant. Only 8 % sought medical intervention and the proportions were not significantly different by occupation (*p* = 0.690). Also, as described above, when TTP analysis was censored at 14 months [1], the results remained similar.

#### Occupational exposures in domestic work

In different parts of the world, domestic workers are exposed to a myriad of occupational exposures, including chemical, physical and psychosocial exposures, which can cause reproductive harm. This may even be more severe in less developed countries like South Africa where workplace regulation and employee education are minimal or non-existent. The most important of domestic work hazards is exposure to chemical agents. Domestic workers and cleaners are exposed to a number of volatile organic solvents, detergents, disinfectants and other chemical agents used for cleaning purposes in domestic work and other cleaning environments. Some cleaning solvents include halides, hydrocarbons, formaldehyde, alcohols, ketones, aldehyedes, esters, and ethers [22].

A number of chemicals used in domestic work have been implicated in reduced fecundity in other occupational groups [10, 11, 23, 24]. For example, chlorine, surfactants, formaldehyde, perchloroethylene (PERC) and ammonia commonly present in household cleaning products, soaps, air fresheners, scouring powders, etc., have been shown to be associated with increased time-to-pregnancy and pregnancy loss [10, 25]. A Finnish occupational study found that formaldehyde, which is commonly used in household cleaning substances, was associated with decreased fecundity: a fecundability ratio of 0.64 (95 % CI 0.43–0.92) was found in women with high levels of formaldehyde exposure in comparison to an unexposed group. A higher odds of endometriosis (OR = 4.5; 95 % CI 1–20) was also reported in the study [25].

For many of these substances, inhalation is the primary route of exposure, although dermal exposure is also important. Sadly, there are no regulations for the chemical contents in household products. While an average individual might use these products periodically, domestic workers use them routinely and in combinations. Such routine exposures can be frequent and chronic. Other chemical exposures could come from secondhand and thirdhand smoke. While most workplaces have banned smoking in South Africa, in private residences it is not banned. This can expose domestic workers to hazardous compounds from tobacco smoke. Acrolein, furan, acrylonitrile, and 1,3-butadiene are some of the most harmful volatile organic compounds to be identified in tobacco smoke residue [26].

In addition, domestic workers may be more exposed to physical and psychological stressors than administrative workers. Physical stressors such as awkward working postures, long working hours and physical exhaustion are common in domestic work, and these factors have been implicated in reduced fecundity. A study of the determinants of pregnancy outcome found that physical exertion in domestic work is associated with adverse outcomes [27]. Very early pregnancy loss, before its detection, can lead to prolonged time-to-pregnancy. Poor working conditions, high workload, poor remuneration and lack of developmental prospects can result in psychosocial stress for domestic workers. Domestic workers usually have limited skills for employment in formal sectors. This often means that they have to accept and work in any kind of environment domestic work presents, leading to psychosocial stress that can impact reproductive and overall health. Some studies have reported the role of psychosocial stress on increased TTP [28, 29].

### External validity

Although the overall reproductive health study was representative of the study population, the women analysed in this TTP study have different characteristics from the overall sample. This is largely driven by the fact that pregnancy planning is low in South Africa and women who plan their pregnancy are different from those who do not, at least in terms of some basic socio-demographic characteristics. It is important to consider that the generalisability of these findings to all domestic workers in the population may be easily violated by the selective force of planning. It is not known to which extent those who participated in the study represent their occupational groups in the overall population. Therefore, external validity of the findings should be interpreted with caution.

However, the reduced sample size in the TTP analysis would be expected to be the case for TTP studies in many settings in South Africa as pregnancy planning is generally low in the population. In 2003, the South Africa Demographic and Health Survey which is a representative survey of the South African population reported a planned pregnancy rate of 50 % for the population [30]. Interestingly, low proportions of planned pregnancy have also been reported for some European countries: 37 % in Poland [31] and 41 % in East Germany [32]. Also, there is no reason to believe that the occupational groups in this study would have significantly different occupational exposure profiles from similar groups in other settings in South Africa.

### Recommendations

Domestic workers may not know enough about the potential hazards of chemical exposure, and as a result they may not ask for or wear personal protection equipment when necessary. It is important for domestic workers to be educated about the potential hazards associated with chemical substances they use in their daily work, the need for safe handling and use of personal protective equipment. Employers should be key players in protecting the health of domestic workers. Also, the government should consider legislating that employers should provide domestic workers with training and protection equipment. Domestic workers’ unions and the government’s domestic workers Skills Development Project can be used as a platform for such training.

The rate at which new chemicals, designed for different purposes, are introduced into the market is high. Most of the chemicals are not tested for their toxicological or epidemiological effects. Simple epidemiological studies which compare exposed and unexposed groups, using data collected by short validated questionnaire, can be useful in identifying quantifiable risks to these agents. The advantage of detecting small increases in average TTP is that the reproductive hazard may be identified and controlled before irreversible damage occurs [33].

While focus on chemical exposure should be primary, physical and psychosocial hazards should also be curtailed. Domestic workers are some of the lowest paid employees in South Africa (median salary = ZAR 1,000) [33]. While salary increase negotiation can be knotty and not always possible, employers can ensure that they create good working conditions for their staff, which will go a long way in reducing the physical and psychosocial stress associated with domestic work. Recently, the South African government has taken actions to improve the working conditions and wage of domestic workers and to protect them. This includes setting rules and regulations for; their working conditions, minimum wage, housing conditions and so on. For example, according to recent regulations, domestic workers should not work more than 45 h a week, and should not work more than 15 h of overtime in a week. While these actions are welcomed, more work is still needed, especially in the area of health protection at work.

## Conclusions

The crude and adjusted analysis showed reduced per-cycle probability of conception in domestic workers with reference to administrative staff. As this was a cross-sectional study, it is not known the extent to which confounding or bias may have played a role in the findings. However, the adjusted analysis remained similar and exploration for common biases did not change the conclusion. Also, the results agree with previous results from the same setting. We believe there are plausible reasons that domestic work may expose women to reproductive injury. However, more studies are needed to confirm this finding, in particular and to investigate TTP and occupation among African women in general.
